# Early-Onset Neonatal Infection and Epilepsy in Children

**DOI:** 10.1001/jamanetworkopen.2025.19090

**Published:** 2025-07-07

**Authors:** Mads Andersen, Niels Bjerregård Matthiesen, May Murra, Stine Yde Nielsen, Tine Brink Henriksen

**Affiliations:** 1 Department of Pediatrics and Adolescent Medicine, Aarhus University Hospital, Aarhus, Denmark; 2 Department of Clinical Medicine, Aarhus University, Aarhus, Denmark; 3 Department of Pediatrics and Adolescent Medicine, Randers Regional Hospital, Randers, Denmark; 4 Department of Clinical Microbiology, Lillebaelt Hospital, Vejle, Denmark; 5 Department of Biomedicine, Aarhus University, Aarhus, Denmark

## Abstract

**Importance:**

Epilepsy is a common neurological disorder in childhood. It remains unclear whether early-onset sepsis is associated with epilepsy and to what extent early-onset meningitis may contribute to its development.

**Objective:**

To study the association between early-onset neonatal infection and childhood epilepsy.

**Design, Setting, and Participants:**

Nationwide, population-based, register-based cohort study of all Danish live-born singletons with at least 35 completed gestational weeks at birth without major congenital anomalies in Denmark between 1997 and 2013 with follow-up until 2021 or age 18 years, whichever came first. Data were analyzed from February 2024 to January 2025.

**Exposures:**

Early-onset infection was defined as an invasive bacterial infection during the first week of life. Diagnosed infection was defined by diagnostic codes of sepsis or meningitis, while culture-positive infection only included those with bacterial pathogens cultured from blood or cerebrospinal fluid.

**Main outcomes and measures:**

Epilepsy was defined as a hospital diagnosis or at least 2 redeemed prescriptions of antiepileptic medication. The association between diagnosed sepsis and epilepsy was investigated with multivariable Cox regression to estimate adjusted hazard ratios (HR) with 95% CIs. Associations for the remaining definitions of infection were investigated using person-time incidence rate ratios (IRR).

**Results:**

Among 981 869 children, the median (IQR) gestational age was 40 (39-41) weeks, 501 615 (51%) were male, 8154 (0.8%) were diagnosed with sepsis, and 152 (<0.1%) were diagnosed with meningitis. Of these, 257 children had culture-positive sepsis and 32 had culture-positive meningitis. The incidence rate of epilepsy was 1.6 per 1000 person-years for children with diagnosed sepsis compared with 0.9 per 1000 person-years for children without an infection, resulting in an adjusted HR of 1.85 (95% CI, 1.60-2.13). Culture-positive sepsis was also associated with an increased risk of epilepsy (IRR, 2.70; 95% CI, 1.08-5.56), while the highest risks were observed in children with meningitis both diagnosed (IRR, 9.85; 95% CI, 5.52-16.27) and verified by culture (IRR, 16.04; 95% CI, 5.21-37.46).

**Conclusions and relevance:**

In this nationwide cohort study, early-onset neonatal sepsis was associated with an approximately 2-fold increased risk of childhood epilepsy. As expected, early-onset meningitis was associated with an even higher risk of epilepsy with an approximately 10-fold increase.

## Key Points

**Question:**

Are invasive bacterial infections during the first week of life associated with an increased risk of childhood epilepsy?

**Findings:**

In this nationwide cohort study of 981 869 children, early-onset neonatal sepsis and meningitis, defined by both diagnoses and bacterial cultures, were associated with an increased risk of childhood epilepsy.

**Meaning:**

These findings suggest that improved prevention and treatment of early bacterial infections may help reduce the risk of childhood epilepsy.

## Teaser

This cohort study evaluates the association between early-onset neonatal infection and childhood epilepsy.

## Introduction

Early-onset neonatal infection may be defined as an invasive bacterial infection that occurs within the first week of life, including conditions such as sepsis and meningitis. The bacteria may be transferred from mother to child through ascending infection before birth or by infection during delivery.^1^ Group B *Streptococcus* (GBS) and *Escherichia coli* remain the most prevalent bacteria in high-income settings.^2,3^ However, most cases of early-onset infections are diagnosed based on clinical symptoms and biochemical markers.^4^

While the mortality rate of early-onset infection is relatively low among full-term infants particularly in high-income countries, bacterial infection remains a major contributor to neonatal deaths globally.^5,6,7^ However, the long-term complications for survivors are unclear.^7,8,9^ Recent literature highlighted the need for further studies to justify the consideration of new preventive strategies and treatments such as maternal vaccination.^8,9,10^

Epilepsy is defined as a neurological disease characterized by an enduring predisposition to generate epileptic seizures and by the neurobiologic, cognitive, psychological, and social consequences of the condition.^11,12^ Epilepsy is a common disease during childhood and adolescence with an estimated lifetime prevalence of up to 1.0% from age 0 to 18 years.^13^ The disease contributes significantly to disability-adjusted life years and increases the risk of premature death and mental health issues.^14,15,16^ In addition, epilepsy imposes a considerable economic strain on both the individual and the society.^17^ This emphasizes the need for research into preventable underlying causes. Epilepsy may arise from various causes including genetic, structural, metabolic, immunological, and infectious.^18^ Among infectious causes, the focus in children has mostly been on central nervous system infections such as meningitis, whereas the association with sepsis, a far more common infection, remains insufficiently explored.^19,20,21,22,23^

We hypothesize that the local and systemic inflammation caused by both early-onset sepsis and meningitis may lead to cerebral injury and interfere with normal brain development, increasing the risk of neurological disorders such as epilepsy.^8^ We conducted a nationwide register-based cohort study in near-term and full-term children, aiming to investigate the association between early-onset neonatal infection, defined by both diagnoses and bacterial cultures, and childhood epilepsy.

## Methods

### Study Population

This nationwide register-based cohort study included all live-born singleton infants in Denmark from 1997 to 2013 born after 35 completed gestational weeks. Children with major congenital anomalies were excluded.^7^ The population was identified in the Danish Medical Birth Register.^24^ Information from the various Danish registers were linked to each child using a unique personal identifier assigned to all those residing in Denmark.^25^ An overview of the data sources is provided in eMethods 1 in Supplement 1. The study was approved by the Central Denmark Region and Statistics Denmark. No ethical approval or informed consent were required as the study was based on deidentified registry data. The study was reported in accordance with the Strengthening the Reporting of Observational Studies in Epidemiology (STROBE) reporting guideline.^26^

### Early-Onset Infection

Early-onset neonatal infection was defined as an invasive bacterial infection with onset during the first week of life. Infections were categorized into diagnosed and culture-positive sepsis and meningitis (Table 1). Children with both sepsis and meningitis were categorized as having meningitis only. Diagnosed infection was defined by diagnostic codes of sepsis and meningitis according to the *International Statistical Classification of Diseases and Related Health Problems, Tenth Revision (ICD-10)*, based on the neonatal sepsis and meningitis guidelines from the Danish Pediatric Society (Table 1).^27^ This information was obtained from the Danish National Patient Register.^28^ The duration of admission was required to be at least 5 days, unless the child died earlier. This aimed to exclude children in whom intravenous antibiotics were discontinued early due to rebutted suspicion of infection. A subgroup of children with diagnosed infection also had a culture-positive infection. These children had a bacterial pathogen cultured from blood or cerebrospinal fluid (Table 1). Information on bacterial cultures was obtained from the Danish Departments of Clinical Microbiology from 2000 to 2013.

**Table 1.** Study Definitions of Diagnosed and Culture-Positive Sepsis and Meningitis Within the First Week of Life

Diagnosed infection	*ICD*-*10*	Culture-positive infection^a^
Sepsis		
Streptococcal sepsis	DA40.0-DA40.9	Culture of bacterial pathogen from the blood.
Other sepsis	DA41.0-DA41.9	
Bacterial pneumonia	DJ15.0-DJ15.9	
Congenital pneumonia	DP23.1-DP23.9	
Bacterial sepsis of newborn	DP36.0-DP36.9	
Meningitis		
Bacterial meningitis	G00.0-G00.9	Culture of bacterial pathogen from the cerebrospinal fluid.
Meningitis, unspecified	G03.9	

Abbreviation: *ICD-10*, *International Statistical Classification of Diseases and Related Health Problems, Tenth Revision*.

^a^ Pathogens included Enterobacterales (eg, *Escherichia coli* and *Klebsiella spp*), *Enterococcus faecalis*, *Enterococcus faecium*, *Haemophilus influenzae*, *Listeria monocytogenes*, *Pseudomonas aeruginosa*, *Staphylococcus aureus*, Group B *Streptococcus*, *Streptococcus dysgalactiae*, *Streptococcus pneumoniae*, and *Streptococcus pyogenes*.

### Epilepsy

Epilepsy was defined as a hospital diagnosis or at least 2 redeemed prescriptions of antiepileptic medication. Diagnoses included primary *ICD-10* codes of epilepsy obtained from the Danish National Patient Register (DG40).^28^ *ICD-10* codes were also used to identify subtypes of epilepsy including focal epilepsy (DG40.1 and DG40.2) and generalized epilepsy (DG40.3). Prescriptions were identified based on the anatomical therapeutic chemical codes obtained from the Danish National Prescription Register including antiepileptics (N03A) and clobazam (N05BA09) with exception of gabapentin (N03AX12).^29^

### Potential Confounding Variables

Potential confounding variables were illustrated by directed acyclic graphs (eFigure 1 in Supplement 1).^30^ The variables were categorized as shown in Table 2. These included child characteristics such as sex, gestational age, birth weight, and birth year; maternal characteristics such as age, parity, smoking during pregnancy, and diabetes; and socioeconomic factors determined at the year of birth such as ethnicity, highest parental education, family disposable income, and parental cohabitation. Maternal diabetes was defined as a diagnosis of diabetes or gestational diabetes or at least 2 prescriptions of antidiabetic medication before delivery. This information was obtained from the Danish National Patient Register and the Danish National Prescription Register.^28,29^ Information on ethnicity was obtained from Statistics Denmark and the Danish Civil Registration System.^31,32^ Statistics Denmark also provided information on the remaining socioeconomic factors, while the Danish Civil Registration System also provided information on censoring events such as deaths and emigrations.^31,32^

**Table 2.** Characteristics of Children With and Without Diagnosed Early-Onset Infection (Sepsis and Meningitis)^a^

Characteristic	Participants, No. (%)	
	No infection (n = 973 563)	Diagnosed infection (n = 8306)
Child		
Sex		
Female	477 186 (49)	3068 (37)
Male	496 377 (51)	5238 (63)
Gestational age, median (IQR), wk	40 (39-41)	40 (39-41)
Birth weight, mean (SD), g	3560 (510)	3589 (662)
Maternal		
Age, mean (SD), y	30 (4.9)	30 (5.1)
Nulliparous	416 995 (43)	5337 (64)
Smoking during pregnancy	141 524 (15)	1314 (16)
Diabetes	30 165 (3)	386 (5)
Socioeconomics		
Western origin^b^	857 527 (88)	7326 (88)
Parental education		
Low	463 407 (48)	4170 (50)
Medium	295 651 (30)	2472 (30)
High	214 505 (22)	1664 (20)
Yearly family income, median (IQR), 1000 DKK	188 (145-234)	188 (143-231)
Parental cohabitation	852 585 (88)	6995 (84)

Abbreviation: DKK, Danish Krone.

^a^ The study included near-term and full-term children born in Denmark from 1997 to 2013.

^b^ Maternal birthplace or citizenship in countries within the European Union and other countries including Andorra, Great Britain, Iceland, Lichtenstein, Monaco, Norway, San Marino, Switzerland, Vatican State, Canada, US, Australia, and New Zealand.

### Statistical Analyses

The children were followed up until the earliest occurrence of either a hospital diagnosis of epilepsy or 2 prescriptions of antiepileptic medication, or a censoring event such as death, emigration, turning 18 years old, or June 30, 2021. Incidence rates and incidence rate ratios (IRR) were estimated for children with and without early-onset sepsis and meningitis. Multivariable Cox regression analysis was then conducted to estimate adjusted hazard ratios (HR) between children with and without diagnosed sepsis. This analysis was considered the primary analysis. Multivariable Cox regression was also used to estimate the association between diagnosed sepsis and focal and generalized epilepsy. As the proportional hazards assumption was violated, Cox regression was not used to evaluate the associations involving meningitis and culture-positive infection. All estimates were provided with 95% CIs. Missing values were handled by multiple imputation (eMethods 2 in Supplement 1). Statistical analyses were performed using Stata version 18 (StataCorp LLC) from February 2024 to January 2025.

The robustness of the results from the primary analysis was evaluated by several sensitivity analyses. Complete-case analysis was conducted excluding all children with missing values. The dependence between children with the same mother was considered by cluster analysis with robust standard errors. To evaluate any potential misclassification of epilepsy, analysis was also conducted with epilepsy requiring both a diagnosis and 2 prescriptions of antiepileptic medication. Early-onset infection may also be defined as an infection occurring within the first 3 days of life.^33^ An analysis was therefore conducted considering sepsis presenting within this time period only. Ascending bacteria that cause infection in utero are plausible contributors to asphyxia and low Apgar score at birth. However, as it also may be difficult to distinguish between birth asphyxia and early-onset sepsis, we conducted an additional analysis excluding children with severe birth asphyxia and 1-minute Apgar scores between 0 and 3 (*ICD-10*: DP21.0). Gestational age and birth weight may confound the association between early-onset infection and epilepsy, but ascending infections may also cause preterm birth.^34,35^ An additional analysis was therefore also conducted excluding these variables from the primary analysis. To evaluate any unmeasured shared familial confounding, sibling-matched analysis was also conducted using stratified Cox regression including children with the same mother. Significance was determined as 95% CIs not containing 1.

## Results

### Population Characteristics

A total of 981 869 near-term and full-term children were included in the study. The median (IQR) gestational age was 40 (39-41) weeks and 501 615 (51%) were male. A total of 8154 children (0.8%) were diagnosed with sepsis, while 152 children (<0.1%) were diagnosed with meningitis. A culture-positive infection was found in 257 children (<0.1%) with diagnosed sepsis and in 32 (<0.1%) with diagnosed meningitis. The most common diagnosis was unspecified newborn bacterial sepsis (DP36.9) (6839 patients [84%]). The most common bacterial pathogen was GBS (156 patients [54%]) followed by *Staphylococcus aureus* (52 patients [18%]) and *E. coli* (36 patients [12%]). A total of 12 228 children (1.2%) developed epilepsy during the study period, of which 2678 (0.3%) were defined as having focal epilepsy and 2685 (0.3%) as having generalized epilepsy. The median (IQR) age for being recorded with epilepsy was 6 (2-10) years for children with an infection and 7 (3-12) years for children without an infection. Among children with an infection, 49 (23%) were diagnosed within the first year of life, compared with 1463 (12%) in the reference population. A total of 62 204 children (6.3%) were censored before meeting the criteria for epilepsy, with 2276 children (0.2%) dying and 59 928 children (6.1%) emigrating. Among children with diagnosed infection, 123 died (1.5%) compared with 2153 (0.2%) in the reference group. A flowchart of the study population is provided in eFigure 2 in Supplement 1. Baseline characteristics of children with and without diagnosed infection are shown in Table 2, while characteristics of children with culture-positive infection are shown in eTable 1 in Supplement 1.

### Diagnosed Sepsis

Incidence rates and IRR for children with and without diagnosed sepsis are shown in Table 3, while an overview of the results from the primary analysis is provided in Table 4. The incidence rate of epilepsy for children with diagnosed sepsis was 1.6 per 1000 person-years, compared with 0.9 per 1000 person-years for children without infection. Children with diagnosed sepsis had an increased risk of epilepsy following adjustment for potential confounders (HR, 1.85; 95% CI, 1.60-2.13). Diagnosed sepsis was associated with both focal epilepsy (HR, 2.94; 95% CI, 2.30-3.75) and generalized epilepsy (HR, 1.46; 95% CI, 1.05-2.05). No major difference was seen between the adjusted and unadjusted estimates.

**Table 3.** Associations Between Various Definitions of Early-Onset Infection and Childhood Epilepsy^a^

Type of infection	Participants, Total No.	Epilepsy events, No. (%)	IR per 1000 y (95% CI)	Person-time, IRR (95% CI)
No infection (1997-2013)	973 563	12 011 (1.2)	0.9 (0.9-0.9)	1.00 [Reference]
Diagnosed sepsis	8154	194 (2.4)	1.6 (1.4-1.9)	1.89 (1.63-2.18)
Diagnosed meningitis	152	15 (9.9)	8.6 (5.2-14.2)	9.85 (5.52-16.27)
No infection (2000-2013)	800 188	9201 (1.1)	0.8 (0.8-0.9)	1.00 [Reference]
Culture-positive sepsis	257	7 (2.7)	2.3 (1.1-4.8)	2.70 (1.08-5.56)
Culture-positive meningitis	32	5 (15.6)	13.5 (5.6-32.4)	16.04 (5.21-37.46)

^a^ The study included near-term and full-term children born in Denmark from 1997 to 2013 (diagnosed infection) or 2000 to 2013 (culture-positive infection).

**Table 4.** Associations Between Diagnosed Early-Onset Sepsis and Childhood Epilepsy^a^

Epilepsy type	Participants, No. (%)		Unadjusted HR	Adjusted HR (95% CI)
	Epilepsy within reference group	Epilepsy following diagnosed sepsis		
All epilepsy	12 011 (1.2)	194 (2.4)	1.89	1.85 (1.60-2.13)
Focal	2612 (0.3)	66 (0.8)	3.02	2.94 (2.30-3.75)
Generalized	2650 (0.3)	35 (0.4)	1.50	1.46 (1.05-2.05)

^a^ The study included near-term and full-term children born in Denmark from 1997 to 2013. A total of 973 563 children were included in the reference group, while 8154 children were diagnosed with sepsis. The multivariable model included: child sex, gestational age, birth weight, birth year, maternal age, parity, maternal smoking, maternal diabetes, ethnicity, parental education, family disposable income, and parental cohabitation.

### Culture-Positive Sepsis and Meningitis

An overview of the associations between the various definitions of infection and childhood epilepsy is summarized in Table 3. The incidence rate of epilepsy in children with culture-positive sepsis was 2.3 per 1000 person-years. This resulted in an increased risk of epilepsy for children with culture-positive sepsis (IRR, 2.70; 95% CI, 1.08-5.56). The incidence rates were even higher for children with meningitis, with 8.6 per 1000 person-years for diagnosed meningitis and 13.5 per 1000 person-years for culture-positive meningitis. This also resulted in an increased risk of epilepsy following meningitis, defined both by diagnoses (IRR, 9.85; 95% CI, 5.52-16.27) and verified by culture (IRR, 16.04; 95% CI, 5.21-37.46). The absolute number of children who developed epilepsy was considerably higher after sepsis compared with meningitis (194 vs 15 children).

### Results From Sensitivity Analyses

None of the sensitivity analyses yielded results that were substantially different from those of the primary analyses. The association between diagnosed sepsis and epilepsy was slightly more pronounced when epilepsy required both a diagnosis and at least 2 prescriptions of antiepileptic medication (HR, 2.22; 95% CI, 1.85-2.66). This was similar to the association observed between siblings with and without diagnosed sepsis (HR, 2.07; 95% CI, 1.55-2.79). However, when excluding children with severe asphyxia and low Apgar score at birth, the adjusted hazard ratio was slightly reduced to 1.62 (95% CI, 1.38-1.90). Redefining early-onset sepsis to occur within the first three days of life as well as the other sensitivity analyses revealed estimates more closely resembling those from the primary analysis (eTable 2 in Supplement 1).

## Discussion

In this Danish nationwide cohort study, we found that both diagnosed and culture-positive early-onset sepsis was associated with an approximately 2-fold increased risk of childhood epilepsy. The association between diagnosed sepsis and epilepsy was more pronounced for focal epilepsy compared with generalized epilepsy. Children with early-onset meningitis faced an even higher risk of epilepsy, with an approximately 10-fold increase compared with children without infection.

Unlike the well-described increased risk of epilepsy following childhood meningitis, the association between early-onset sepsis and epilepsy has not been thoroughly examined.^8,9,19,20,21,22^ This is despite sepsis being a far more common disease that potentially may contribute substantially more to the overall prevalence of epilepsy.^23^ This was supported by our findings as the absolute number of children who developed epilepsy was considerably higher after sepsis compared with meningitis (194 vs 15 children). Meningitis may cause brain injuries by several mechanisms including cerebral inflammation and cytotoxicity as well as secondary effects such as brain edema and increased intracranial pressure.^8^ However, the systemic inflammation caused by early-onset sepsis may also involve the central nervous system. The recognition of pathogens by toll-like-receptors and secretion of cytokines may disrupt the blood-brain barrier and result in microglia activation and neuronal cell death.^8,36,37,38^ The systemic inflammation triggered by sepsis may also lead to hypotension and cerebral hypoperfusion. These effects may altogether lead to structural damage of the developing brain. Our results could potentially be consistent with this, finding that early-onset sepsis was more associated with focal than generalized epilepsy. Generally, structural brain lesions are considered to be more likely to result in focal epilepsy than in generalized epilepsy, while generalized epilepsy primarily is attributed to a genetic predisposition.^39,40^ Another Danish cohort study did not find an increased risk of epilepsy comparing children with and without *ICD-10* codes of GBS sepsis from 0 to 89 days of life.^22^ This contrasts with our findings, showing an almost doubling of the risk of epilepsy following diagnosed sepsis. This discrepancy could be explained by differences in study designs. We only focused on infections occurring within the first week of life. Invasive bacterial infections may have a greater impact on the brain during the earliest stage of life, when both the brain and the immune system are less mature. Additionally, we included diagnoses of other pathogens than GBS as well as unspecified diagnoses of newborn sepsis. Notably, unspecified newborn bacterial sepsis (DP36.9) represented the largest proportion of diagnosed sepsis in our study. However, our analysis restricted to children with culture-positive sepsis also revealed an increased risk of epilepsy, with these children showing a higher point estimate compared with those with diagnosed sepsis.

### Limitations

We conducted a large population-based study of near-term and full-term children investigating the association between early-onset infection, defined by both diagnoses and bacterial cultures, and epilepsy with long-term follow-up throughout childhood. This study therefore addresses several needs previously emphasized by other reviews and meta-analyses.^8,9^ However, some limitations should be noted as well. Due to the lack of a consensus definition for neonatal sepsis, some children with diagnosed sepsis may have been misdiagnosed, especially as the infections often present with nonspecific clinical symptoms and biochemical markers without the pathogen being identified.^33^ For this reason, we also restricted our analyses to children with culture-positive sepsis. Bacteria-specific analyses were unfortunately impossible due to the small number of children with a verified pathogen. We also performed a sensitivity analysis excluding children with severe asphyxia and low Apgar scores at birth, a condition that potentially could lead to a misdiagnosis of sepsis. This exclusion yielded results similar to the primary analysis, although with slightly reduced point estimates. However, ascending bacteria and intrauterine infection may also contribute to birth asphyxia and may sensitize the fetal or neonatal brain, exacerbating the cerebral injury following a secondary insult such as hypoxia-ischemia.^41,42^ The observed reduction may therefore reflect the removal of a mediating effect instead of a potential confounding effect.

We used both diagnoses and prescriptions of medication to identify children with epilepsy. The diagnoses of epilepsy from the Danish National Patient Register generally show a moderate to high positive predictive value when compared with the clinical information from the patient medical records.^28,43^ A previous systematic review found that combining *ICD-10* codes of epilepsy with antiepileptic medication may increase the positive predictive value.^44^ The risk of epilepsy was only slightly more pronounced in our sensitivity analysis with epilepsy requiring both, suggesting only a minor degree of outcome misclassification. However, the validity of specific subtypes of epilepsy has been found more questionable, which means that our analyses of focal and generalized epilepsy should be interpreted with caution.^43,45^

Our dataset was essentially complete with maternal smoking being the covariate with most missing values (3.4%). Multiple imputation was used to account for missing values with inclusion of several auxiliary variables. The loss to follow-up in the study was therefore solely due to deaths or emigrations. The mortality was almost 8 times higher in children with early-onset infection than in children without. We are therefore unable to rule out any potential survivor bias and may have underestimated the associations if those who died would have had a higher risk of epilepsy.

Several potential confounders were integrated in our multivariable model including both child, maternal, and socioeconomic factors. Despite this extensive inclusion, we observed no meaningful differences between the adjusted and unadjusted risks. Some forms of epilepsy may arise from genetic factors, which also may have confounded the observed associations.^39,40^ However, the sibling-matched analysis showed results consistent with the primary analysis. This suggests that unmeasured shared familial confounding, such as shared genetics, is unlikely to have introduced bias. Additionally, cluster analysis resulted in similar variance as the primary analysis, suggesting that the assumption of independence between children of the same mother was reasonable.

This study was conducted in a high-income country, limiting its generalizability to low- and middle-income countries. Early-onset infection may be associated with an even higher risk of epilepsy in resources-limited settings due to suboptimal newborn care. As previously suggested, additional research is needed to investigate the long-term effects of early bacterial infections in various settings to improve our understanding of the global impact of the disease.^8^

## Conclusions

This nationwide cohort study of near-term and full-term children found an association between early-onset neonatal infection, defined by both diagnoses and bacterial cultures, and childhood epilepsy. Early-onset sepsis was associated with an approximately 2-fold increased risk. As expected, early-onset meningitis was associated with an even higher risk with an approximately 10-fold increase.

## Notes

Supplement 1. **eMethods 1. **Data Sources **eFigure 1. **Directed Acyclic Graph **eMethods 2. **Overview of the Multiple Imputation **eFigure 2. **Flowchart of the Study Population **eTable 1. **Characteristics of Children With Culture-Positive Infection **eTable 2. **Results From Sensitivity Analyses
eReferences

Supplement 2.
Data Sharing Statement
