# Supplementary material for: Early-Onset Neonatal Infection and Epilepsy in Children
Source: JAMA Netw Open. 2025 Jul 7;8(7):e2519090. doi: 10.1001/jamanetworkopen.2025.19090 (PMC12235498; doi:10.1001/jamanetworkopen.2025.19090)
Supplement: Supplement 1. — eMethods 1. Data Sources eFigure 1. Directed Acyclic Graph eMethods 2. Overview of the Multiple Imputation eFigure 2. Flowchart of the Study Population eTable 1. Characteristics of Children With Culture-Positive Infection eTable 2. Results From Sensitivity Analyses eReferences [file jamanetwopen-e2519090-s001.pdf]

Supplemental Online Content

Andersen M, Matthiesen NB, Murra M, Nielsen SY, Henriksen TB. Early-onset neonatal infection and epilepsy in children. *JAMA Netw Open*. 2025;8(7):e2519090. doi:10.1001/jamanetworkopen.2025.19090

eMethods 1. Data Sources
eFigure 1. Directed Acyclic Graph
eMethods 2. Overview of the Multiple Imputation
eFigure 2. Flowchart of the Study Population
eTable 1. Characteristics of Children With Culture-Positive Infection
eTable 2. Results From Sensitivity Analyses
eReferences

This supplemental material has been provided by the authors to give readers additional information about their work.

© 2025 Anderson M et al. *JAMA Network Open*.

eMethods 1. Data sources

The Civil Registration System

The Civil Registration System has functioned since 1686. It contains information on all Danish citizens including a unique identifier, parentage and citizenships, and status with date of events such as death and emigration. The unique identifier is a ten-digit number assigned to each Danish citizen. It provides information on birth date and gender and allows for linkage of individual-level information between the various national registers. Data in the register are of high quality, as registration is required by law and used for administrative purposes.[1,2]

Danish Departments of Clinical Microbiology

The Danish Departments of Clinical Microbiology were contacted for information on bacterial cultures including date of sampling, sampling material, and pathogen. A total of nine departments were able to provide information from 2000 to 2013 (Aalborg, Aarhus, Viborg/Herning, Esbjerg, Odense, Roskilde, Køge, Herlev, and Hvidovre), while two departments only had information available from 2002 to 2013 (Vejle and Rigshospitalet).

Danish Medical Birth Register

The Danish Medical Birth Register was established in 1973. It contains information on all births in Denmark by women with Danish residency. This includes information on both the mother, pregnancy, and child. Most data are believed to be of high quality with high completeness, as many variables are mandatory to report.[3]

Danish National Patient Register

The Danish National Patient Register was established in the 1977. It registers all hospital contacts including admissions, emergencies, and ambulatory visits. This includes primary and secondary diagnoses, date of admission, and date of discharge. Diagnoses have been registered according to the International Classification of Diseases 10th revision since 1994.[4,5]

Danish National Prescription Register

The Danish National Prescription Register has since 1994 registered all prescription drugs dispensed at Danish pharmacies. It contains information on the dispensed drug and the dispensing date. The data are believed to be of high quality.[6]

Statistics Denmark

Statistics Denmark is the central authority of Danish statistics.[7] It manages several registers containing variables, which were used in study. Variables included ethnicity, highest parental education, family disposable income, and parental cohabitation. All study data were stored and analyzed in servers from Statistics Denmark.

© 2025 Anderson M et al. *JAMA Network Open*.

eFigure 1. Directed Acyclic Graph

A Directed Acyclic Graph illustrating potential confounding variables on the association between early-onset infection and childhood epilepsy. The graph was made by www.dagitty.net (accessed 07.03.2024).

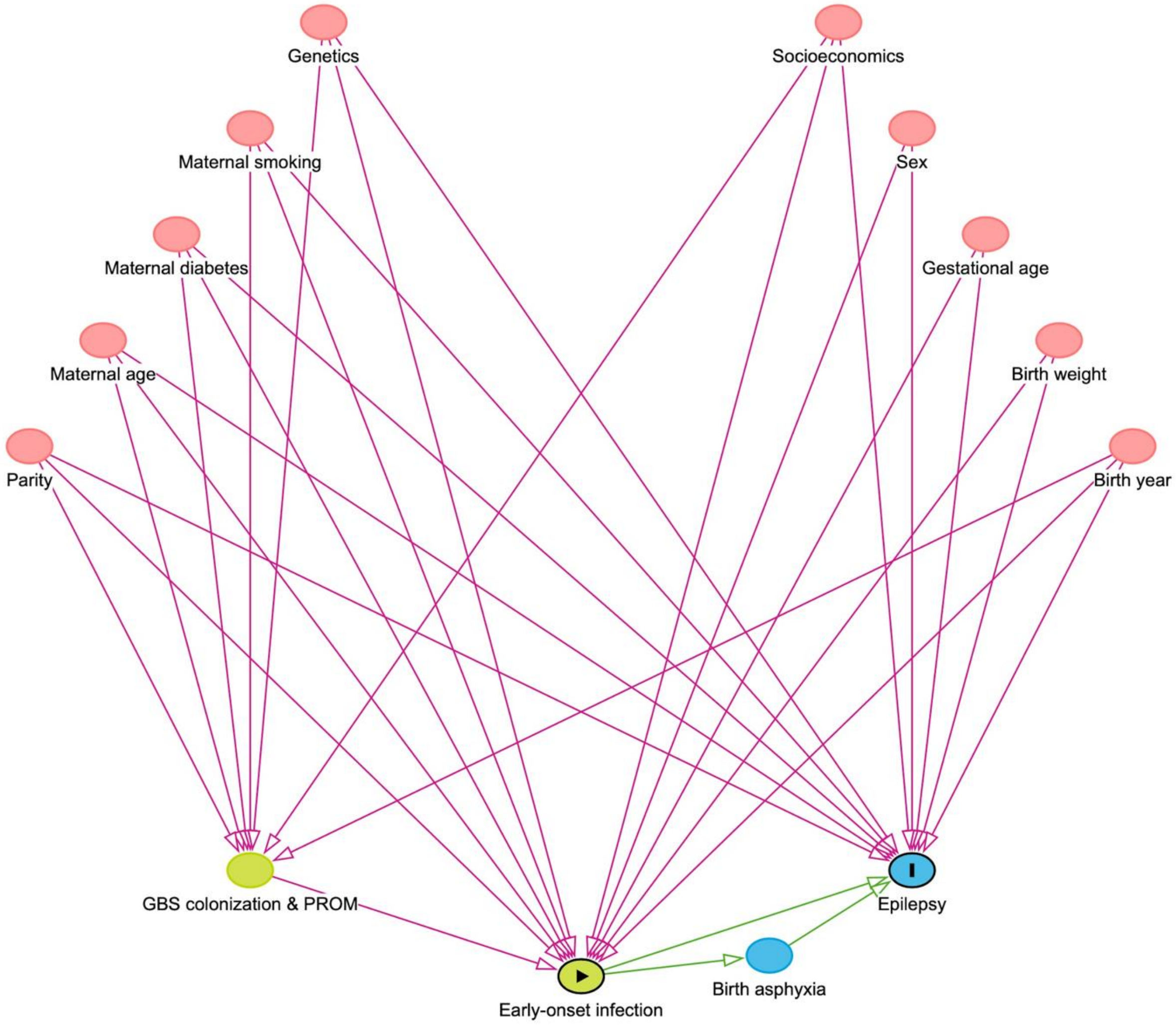

Socioeconomic factors included ethnicity, parental education, family disposable income, and parental cohabitation.

GBS, Group B *Streptococcus*; PROM, premature rupture of membranes

© 2025 Anderson M et al. *JAMA Network Open*.

eMethods 2. Overview of the Multiple Imputation

Three variables were imputed including gestational age (0.9%), birth weight (1.0%), and maternal smoking (3.4%). Chained prediction equations were used including linear and logistic regressions. A total of five imputations with five iterations were used, which was deemed suitable according to Von Hippel 2018.[8] All auxiliary variables were associated with the variables containing missing values or the values being missing (see below). The assumption of missing at random was therefore considered fulfilled.

Related to the mother	Body mass index, psychiatric disease, chronic hypertension, epilepsy, inflammatory disease, and thyroid disorder.
Related to the pregnancy	Home birth, abnormal fetus presentation, pelvic disproportion, placental abruption, placenta accreta, placental insufficiency, placenta previa, polyhydramnios, prolonged rupture of membranes, maternal fever, maternal urinary tract infection during pregnancy, labor induction, vacuum extraction, and preeclampsia.
Related to the child	Head circumference, length, 5-min Apgar score, scalp pH, birth asphyxia, duration of stay in the neonatal intensive care unit, use of continuous positive airway pressure during admission, use of mechanical ventilation during admission, minor congenital anomaly, attention deficit hyperactivity disorder, autism spectrum disorder, mental retardation, psychomotor delay, emigration, death, epilepsy, and baseline hazard for epilepsy.
From the statistical model	Diagnosed infection, culture-positive infection, sex, birth year, maternal age, parity, maternal diabetes, ethnicity, parental education, family disposable income, and parental cohabitation.

All auxiliary variables were obtained from the registers described in eMethods 1.

© 2025 Anderson M et al. *JAMA Network Open*.

eFigure 2. Flowchart of the Study Population

Flowchart showing the number of children at each level of the study.

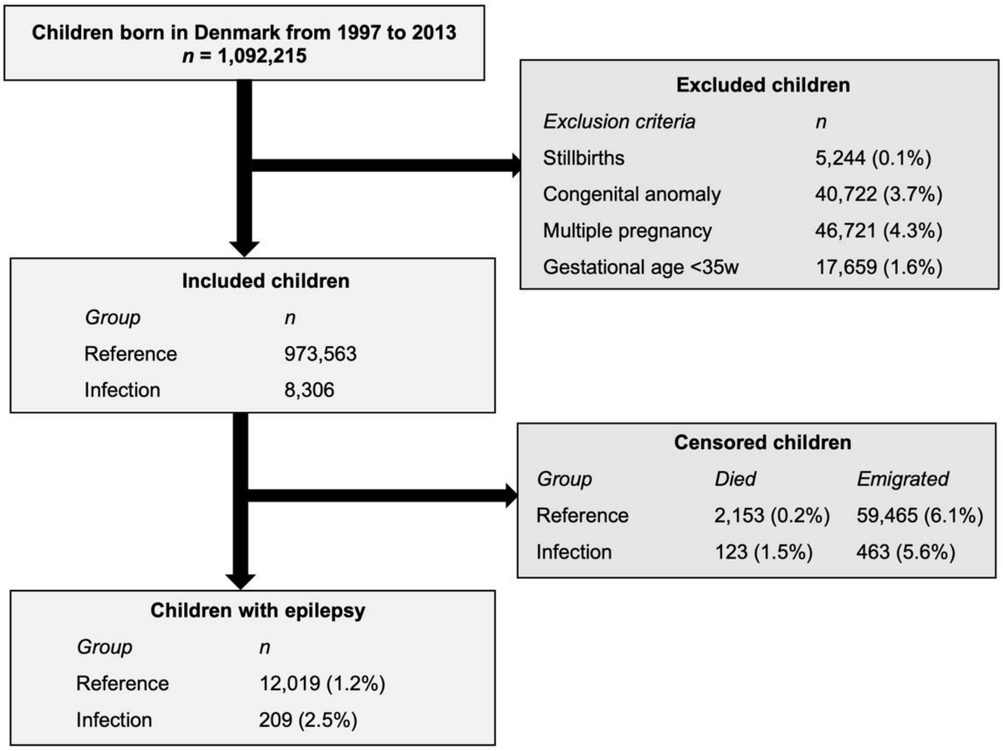

The children were followed until their 18th birthday or June 30, 2021. The median follow-up time was 16 years (interquartile range: 11-18).

© 2025 Anderson M et al. *JAMA Network Open*.

eTable 1. Characteristics of Children With Culture-Positive Infection

Characteristics of near-term and term children with and without culture-positive early-onset infection born in Denmark from 2000 to 2013.

	References (n = 800,188)	Culture-positive infection (n = 289)
**Child characteristics**		
Female	391,583 (49%)	118 (41%)
Gestational age, median (IQR), weeks	40 (39-41)	40 (38-41)
Birth weight, mean (SD), g	3,558 (509)	3,460 (732)
**Maternal characteristics**		
Age, mean (SD), years	31 (4.9)	30 (4.9)
Nulliparous	355,890 (44%)	167 (58%)
Smoking during pregnancy	105,036 (13%)	38 (13%)
Diabetes	20,546 (3%)	17 (6%)
**Socioeconomics**		
Western origin	702,139 (88%)	241 (83%)
Parental education		
Low	364,145 (46%)	141 (49%)
Medium	248,713 (31%)	82 (28%)
High	187,330 (23%)	66 (23%)
Yearly family income, median (IQR), 1,000 DKK	193 (148-240)	188 (140-240)
Parental cohabitation	699,930 (87%)	234 (81%)

Continuous variables are presented as mean values with standard deviations (SD) (normally distributed) or medians with interquartile ranges (IQR) (non-normally distributed), while categorical variables are presented as numbers with percentages.

© 2025 Anderson M et al. *JAMA Network Open*.

eTable 2. Results From Sensitivity Analyses

Sensitivity analyses were conducted for the association between diagnosed sepsis and childhood epilepsy in near-term and term children born in Denmark from 1997 to 2013. The reference group included 973,563 children with 8,154 children being diagnosed with sepsis. The multivariable Cox regression analyses included sex, gestational age, birth weight, birth year, maternal age, parity, maternal smoking, maternal diabetes, ethnicity, parental education, family disposable income, and parental cohabitation.

Complete-case analysis
Adjusted hazard ratios for childhood epilepsy comparing children with and without diagnosed sepsis with exclusion of children with missing values.

Events (%) within reference	*Events (%) within exposed*	*Adjusted HR (95% CI)*
11,499 (1.2%)	182 (2.4%)	1.85 (1.59-2.15)

Cluster analysis
Adjusted hazard ratios for childhood epilepsy comparing children with and without diagnosed sepsis considering dependence between children with the same mother.

Events (%) within reference	*Events (%) within exposed*	*Adjusted HR (95% CI)*
12,011 (1.2%)	194 (2.4%)	1.85 (1.60-2.13)

Analysis with epilepsy requiring both diagnosis and prescriptions
Adjusted hazard ratio for childhood epilepsy comparing children with and without diagnosed sepsis with epilepsy requiring both a diagnosis and at least two prescriptions of antiepileptic medication.

Events (%) within reference	*Events (%) within exposed*	*Adjusted HR (95% CI)*
6,387 (0.7%)	122 (1.5%)	2.22 (1.85-2.66)

Analysis with sepsis defined to occur within three days of life
Adjusted hazard ratio for childhood epilepsy comparing children with and without diagnosed sepsis within the first three days of life.

Events (%) within reference	*Events (%) within exposed*	*Adjusted HR (95% CI)*
12,022 (1.2%)	183 (2.4%)	1.82 (1.57-2.11)

Analysis with exclusion of children with severe birth asphyxia
Adjusted hazard ratio for childhood epilepsy comparing children with and without diagnosed sepsis with exclusion of children with severe birth asphyxia and Apgar score between 0-3 (ICD10: DP21.0).

Events (%) within reference	*Events (%) within exposed*	*Adjusted HR (95% CI)*
11,815 (1.2%)	158 (2.1%)	1.62 (1.38-1.90)

Analysis without gestational age and birth weight
Adjusted hazard ratios for childhood epilepsy comparing children with and without diagnosed sepsis without adjustment for gestational age and birth weight.

Events (%) within reference	*Events (%) within exposed*	*Adjusted HR (95% CI)*
12,011 (1.2%)	194 (2.4%)	1.85 (1.60-2.13)

Sibling-matched analysis
Adjusted hazard ratios for childhood epilepsy comparing siblings with and without diagnosed sepsis.

Events (%) within reference	*Events (%) within exposed*	*Adjusted HR (95% CI)*
82 (1.2%)	126 (2.5%)	2.07 (1.55-2.79)

ICD10, International Classification of Diseases 10th revision

© 2025 Anderson M et al. *JAMA Network Open*.

eReferences

1. Pedersen CB. The Danish Civil Registration System. *Scand J Public Health*. Jul 2011;39(7 Suppl):22-5. doi:10.1177/1403494810387965

2. Schmidt M, Pedersen L, Sorensen HT. The Danish Civil Registration System as a tool in epidemiology. *Eur J Epidemiol*. Aug 2014;29(8):541-9. doi:10.1007/s10654-014-9930-3

3. Bliddal M, Broe A, Pottegard A, Olsen J, Langhoff-Roos J. The Danish Medical Birth Register. *Eur J Epidemiol*. Jan 2018;33(1):27-36. doi:10.1007/s10654-018-0356-1

4. Lynge E, Sandegaard JL, Rebolj M. The Danish National Patient Register. *Scand J Public Health*. Jul 2011;39(7 Suppl):30-3. doi:10.1177/1403494811401482

5. Schmidt M, Schmidt SA, Sandegaard JL, Ehrenstein V, Pedersen L, Sorensen HT. The Danish National Patient Registry: a review of content, data quality, and research potential. *Clin Epidemiol*. 2015;7:449-90. doi:10.2147/CLEP.S91125

6. Kildemoes HW, Sorensen HT, Hallas J. The Danish National Prescription Registry. *Scand J Public Health*. Jul 2011;39(7 Suppl):38-41. doi:10.1177/1403494810394717

7. Statistics Denmark. https://www.dst.dk/en. Accessed May 24, 2024.

8. von Hippel PT. How Many Imputations Do You Need? A Two-stage Calculation Using a Quadratic Rule. *Sociological Methods & Research*. 2020;49(3):699-718. doi:10.1177/0049124117747303

© 2025 Anderson M et al. *JAMA Network Open*.
